# Assessment of psychological pain in suicidal veterans

**DOI:** 10.1371/journal.pone.0177974

**Published:** 2017-05-30

**Authors:** Christopher Reist, Steven Mee, Ken Fujimoto, Vivek Rajani, William E. Bunney, Blynn G. Bunney

**Affiliations:** 1 VA Long Beach Healthcare System, Long Beach, California, United States of America; 2 Department of Psychiatry, University of California Irvine, Irvine, California, United States of America; 3 Applied Innovative Psychiatry, Los Alamitos, California, United States of America; 4 Loyola University, Chicago, IIlinois, United States of America; Southeast University Zhongda Hospital, CHINA

## Abstract

Psychological pain is a relatively understudied and potentially important construct in the evaluation of suicidal risk. Psychological pain also referred to as ‘mental pain’ or ‘psychache’ can be defined as an adverse emotional reaction to a severe trauma (e.g., the loss of a child) or may be associated with an illness such as depression. When psychological pain levels reach intolerable levels, some individuals may view suicide as the only and final means of escape. To better understand psychological pain, we previously developed and validated a brief self-rating 10-item scale, Mee-Bunney Psychological Pain Assessment Scale [MBP] in depressed patients and non-psychiatric controls. Our results showed a significant increase in psychological pain in the depressed patients compared to controls. We also observed a significant linear correlation between psychological pain and suicidality in the depressed patient cohort. The current investigation extends our study of psychological pain to a diagnostically heterogeneous population of 57 US Veterans enrolled in a suicide prevention program. In addition to the MBP, we administered the Columbia Suicide Severity Rating Scale (C-SSRS), Beck Depression Inventory (BDI-II), Beck Hopelessness Scale (BHS), and the Barratt Impulsiveness Scale (BIS-11). Suicidal patients scoring above a predetermined threshold for high psychological pain also had significantly elevated scores on all the other assessments. Among all of the evaluations, psychological pain accounted for the most shared variance for suicidality (C-SSRS). Stepwise regression analyses showed that impulsiveness (BIS) and psychological pain (MBP) contributed more to suicidality than any of the other combined assessments. We followed patients for 15 months and identified a subgroup (24/57) with serious suicide events. Within this subgroup, 29% (7/24) had a serious suicidal event (determined by the lethality subscale of the C-SSRS), including one completed suicide. Our results build upon our earlier findings and recent literature supporting psychological pain as a potentially important construct. Systematically evaluating psychological pain along with additional measures of suicidality could improve risk assessment and more effectively guide clinical resource allocation toward prevention.

## Introduction

Suicide is a serious public health problem and one of the leading causes of death worldwide accounting for an estimated 800,000 deaths annually according to recent statistics from the World Health Organization [1]. In the United States alone more than 42,000 individuals die by suicide every year [2] and rates are increasing [3]. Military suicide rates, normally lower than the general US population, have surpassed that of the civilian population [4] and almost doubled for some military services [5].

Psychological pain is a relatively understudied and potentially important construct for the assessment of suicidal risk. Also referred to as ‘mental pain’ or ‘psychache’, psychological pain can be defined as an emotionally-based aversive feeling that at its extreme can be experienced as torment that may result from a severe emotional trauma (such as the loss of a child) or may be associated with an illness such as depression [5]. Schneidman [6] reviewed hundreds of notes left by completed suicides, and was the first to report a common overarching theme (“I can’t stand the ‘mental pain’ any longer”) suggesting that when psychological pain reaches unbearable levels, individuals view death as a final and only means of escape [7].

A review of ‘mental pain’ in mood disorder patients and studies in clinically heterogeneous patient populations found a significant correlation between high levels of psychological pain and suicidality, independent of psychiatric diagnoses [8]. It is likely that psychological pain alone is only one of many contributing factors. For example, depression, hopelessness and impulsiveness are factors known to increase suicide risk [9–12].

In our original study, we developed and validated a brief 10-item self-rating scale, the Mee-Bunney Psychological Pain Assessment Scale [MBP] [5]. The MBP is a self-report instrument designed for use in diverse clinical settings and can be easily administered by clinicians in a variety of settings. Items are rated on a 5-point Likert Scale and include categories such as chronicity (past 3 months), current psychological pain levels, intensity, tolerance (“how much psychological pain can you tolerate before it becomes unbearable?” and risk (“Do you feel the only way to make the psychological pain stop is to die?). We administered the MBP to 73 major depressive episode (MDE) patients and 96 non-psychiatric controls and showed significantly higher levels of psychological pain in the MDE patients. Further, we demonstrated a significant linear correlation between psychological pain and suicidality. Known groups, content and convergent validity and internal reliability for psychological pain (MBP) were also demonstrated in this study. Convergent validity showed that while the MBP and BDI were related (i.e., scores on these measures converged), the variance in scores was distinct in terms of suicidality. Known groups validity was established by demonstrating that after removing partial contributions of the rating scales, significant group differences remained between MDE patients and controls.

In the current study, we assessed psychological pain in a diagnostically heterogeneous population of suicidal VA patients enrolled in a suicide prevention program. In addition to the MBP, we evaluated depression [(Beck Depression Inventory-BDI-II), impulsiveness (Barratt Impulsiveness Scale-BIS II), hopelessness (Beck Hopelessness Scale-BHS) and suicidality (Columbia Suicide Severity Rating Scale (C-SSRS)]. To explore the relationship between psychological pain and future suicidal behavior we followed patients for 15 months and recorded suicide events. Serious suicide events (SSEs) were identified using the lethality/medical damage subscale of the C-SSRS. Due in part to a potential association with high psychological pain and suicidality [6–8] we hypothesized that the suicidal subjects would score higher on the MBP than major depressive episode (MDE) patients from our previous study [5].

## Methods

Informed Consent: This study was approved by the Veteran’s Affairs Long Beach Healthcare System. All subjects provided written signed consents.

Subjects: Suicidal patients: A total of 57 psychiatric patients (23 outpatients and 34 inpatients) from the Veterans Affairs Long Beach Healthcare System (LBVAHS) meeting criteria were consecutively enrolled in the study. [The sample size was determined using data from our earlier study showing sufficient power for N≥50 [5]]. Patients were referred to the LBVAHS Suicide Prevention Program (SPP) for recent (within 3 months) suicidal ideation and/or behavior. The SPP is a team of clinicians who identify and monitor US Veterans who have previously attempted suicide or who are at risk for suicide.

Inclusion criteria: Males and females between 18–75 years of age receiving treatment at LBVAHS for suicidality.

Exclusion criteria: Acute substance intoxication, inability to fluently read and comprehend the English language and/or other conditions that would prevent an individual’s ability to comprehend and meaningfully complete the assessment instruments. Of those approached to participate, 1 patient was excluded due to lack of English fluency.

Table 1 provides demographics for the 57 suicidal patients. All 57 patients completed the assessments. Psychiatric diagnoses including post-traumatic stress disorder (PTSD), major depressive disorder (MDD), bipolar disorder (BPD), history of substance abuse as well as schizophrenia spectrum. Forty patients had co-occurring disorders.

**Table 1:** Demographics of suicidal patients (N = 57).

Variable	Number of patients
Inpatients	35
Outpatients	22
**Gender**	
Males (%)	52 (91%)
Females (%)	5 (9%)
**Mean age (yrs ± SD)**	
Males	48 ±17
Females	45 ±6
**Race (%)**	
Caucasian	29 (51%)
Black	8 (14%)
Hispanic	13 (23%)
Asian	7 (12%)
**Diagnosis at admission**	
Schizophrenia Spectrum	10 (18%)
BPD^a^, mania	2 (4%)
BPD, depressed	2 (4%)
BPD + PTSD	3 (5%)
BPD	1 (2%)
PTSD^b^	10 (18%)
PTSD + substance-induced psychosis	3 (5)
MDD^c^ + PTSD	10 (18)
MDD + Substance use disorder	14 (25)
MDD + psychosis	2 (4)

^a^BPD, bipolar disorder

^b^PTSD, post-traumatic stress disorder

^c^MDD, major depressive disorder

### Assessments

**Initial visit:** All subjects underwent structured diagnostic interviews with the Mini International Neuropsychiatric Interview Version 5.0.0 [(MINI) for DSM-IV and ICD-10 psychiatric disorders] administered by trained clinicians at in- or outpatient settings. Although not a fully comprehensive DSM-IV diagnostic instrument, the MINI provides reliable assessment of mood and anxiety disorders as well as substance abuse/dependence. It has demonstrated high concordance with the SCID for major depression and represents a reduced time burden for the participant [13]. We administered five scales to all patients: 1) Mee-Bunney Psychological Pain Assessment Scale (MBP) [5]; 2) Columbia-Suicide Severity Rating Scale, Baseline version (C-SSRS), to establish the severity and frequency of suicidal ideation [14]; 3) Beck Depression Inventory-II (BDI-II) [15], 4) Beck Hopelessness Scale (BHS) [16]; 5) Barratt Impulsiveness Scale (BIS-11) [17].

**Mee-Bunney Psychological Pain Scale (MBP):** a 10-item self-report measure allowing for rapid assessment of psychological pain in a general clinical setting using a 5-point Likert scale. The MBP measures frequency (“never” to “always”), intensity (“none” to “high”) and past (prior 3 months) and current psychological pain. Additionally, two items address risk to stop the pain, including dying. We used a pre-determined threshold from our original study (i.e., MBP ≥ 32 representing 0.5 standard deviations above the mean for MDE patients) as a cutoff for high psychological pain[5]. The Cronbach’s alpha (a measure of internal consistency for the MBP) was 0.86 for this study.

Columbia-Suicide Severity Rating Scale, Baseline version (C-SSRS): a standardized structured interview scale to assess suicidal ideation and behavior at the initial visit over the past month and historically.

**BDI-II**: a 21-item self-report measure of depression symptom severity obtained from answer choices on a four-point Likert scale to groups of statements. The BDI-II is revised from a previous version (BDI: Diagnostic and Statistical Manual-DSM-IV) to factor changes in diagnoses. The Cronbach’s alpha was .95 for this study

**BHS**: a 20-item self-report inventory designed to measure three aspects of hopelessness: feelings about the future, loss of motivation, and expectations. It has demonstrated construct validity and reliability with a Cronbach’s alpha = .95 for this study.

**BIS-11** is a questionnaire designed to assess the personality/behavioral construct of impulsiveness. It includes 30 items that are scored to yield six first-order factors (attention, motor, self-control, cognitive complexity, perseverance, and cognitive instability impulsiveness) and three second-order factors (attentional, motor, and non-planning impulsiveness). The Cronbach’s alpha for this study is .87

15-month follow-up: Patients were tracked using electronic medical records within the VA system. Information included provider progress notes related to outpatient (including emergency room visits) and/or inpatient treatments. Data was carefully screened for information relevant to suicidal ideation or behavior. The comprehensive review included screening medical records from the Long Beach VA as well as from additional VA facilities. 56/57 patients continued care at a VA facility for the 15-month follow-up. One patient completed suicide. We evaluated seriousness of suicidal events using the lethality/medical subscale of the C-SSRS [14].

To determine whether suicidal patients in this study scored higher for psychological pain than major depressive episode patients (N = 73) from our previous study [5], we compared MBP scores between the two groups.

## Statistical analysis

We utilized multiple regression analysis, with predictors entered into the model in blocks to examine relationships between the various scales and suicidality. As a starting point, a single predictor was included in the model to establish the baseline contribution of that predictor with respect to suicidality as measured by the C-SSRS. We then built on that model by entering the MBP, which served as the second block of predictors. Doing so, we assessed the additional amount of variance that MBP explained in suicidality on top of each of the other predictors. We could not run a model including all predictors (i.e., BIS, BHS, BDI and MBP) due to the small sample size. Group differences between patients and controls were examined by two-tailed t-tests. As multiple statistical significance tests were performed, the Type I error rate for all analyses was set at .01.

To determine whether suicidal patients rated higher for psychological pain on the MBP compared to patients with a major depressive episode from our earlier study [5] we performed a two-tailed t-test post-hoc to assess the difference in means between the two groups.

## Results

### Inpatients versus outpatients

There were no significant differences between inpatients or outpatients on any of the rating scales although there was a trend towards a difference in depression (BDI) (**Table 2).**

**Table 2:** Comparison of test scores of in- versus outpatients^a^.

Rating Scale	Total patients N = 57	Outpatients N = 22	Inpatients N = 35	Inpatients vs outpatients	^a^p-value
	Mean (SD)	Mean (SD)	Mean (SD)	‘t’ value	
MBP^b^	30.14 (7.43)	28.74 (7.73)	31.09 (7.18)	-1.17	0.25
BIS-II^c^	74.12 (13.01)	74.09(14.14)	74.15 (12.40)	-0.02	0.99
BHS^d^	10.11 (6.90)	10.91 (7.30)	9.56 (6.67)	0.72	0.47
BDI-II^e^	13.23 (7.82)	10.48 (7.52)	15.09 (7.57)	-2.26	0.03
C-SSRS^f^	15.19 (5.00)	14.22 (5.15)	15.85 (4.86)	-1.22	0.23

^a^As multiple statistical significance tests were performed, the Type I error rate for all analyses was set at .01.

^b^Mee-Bunney Psychological Pain Scale

^c^Barratt Impulsiveness Scale

^d^Beck Hopelessness Scale

^e^Beck Depression Inventory II

^f^Columbia Suicide Severity Rating Scale

**Fig 1:**
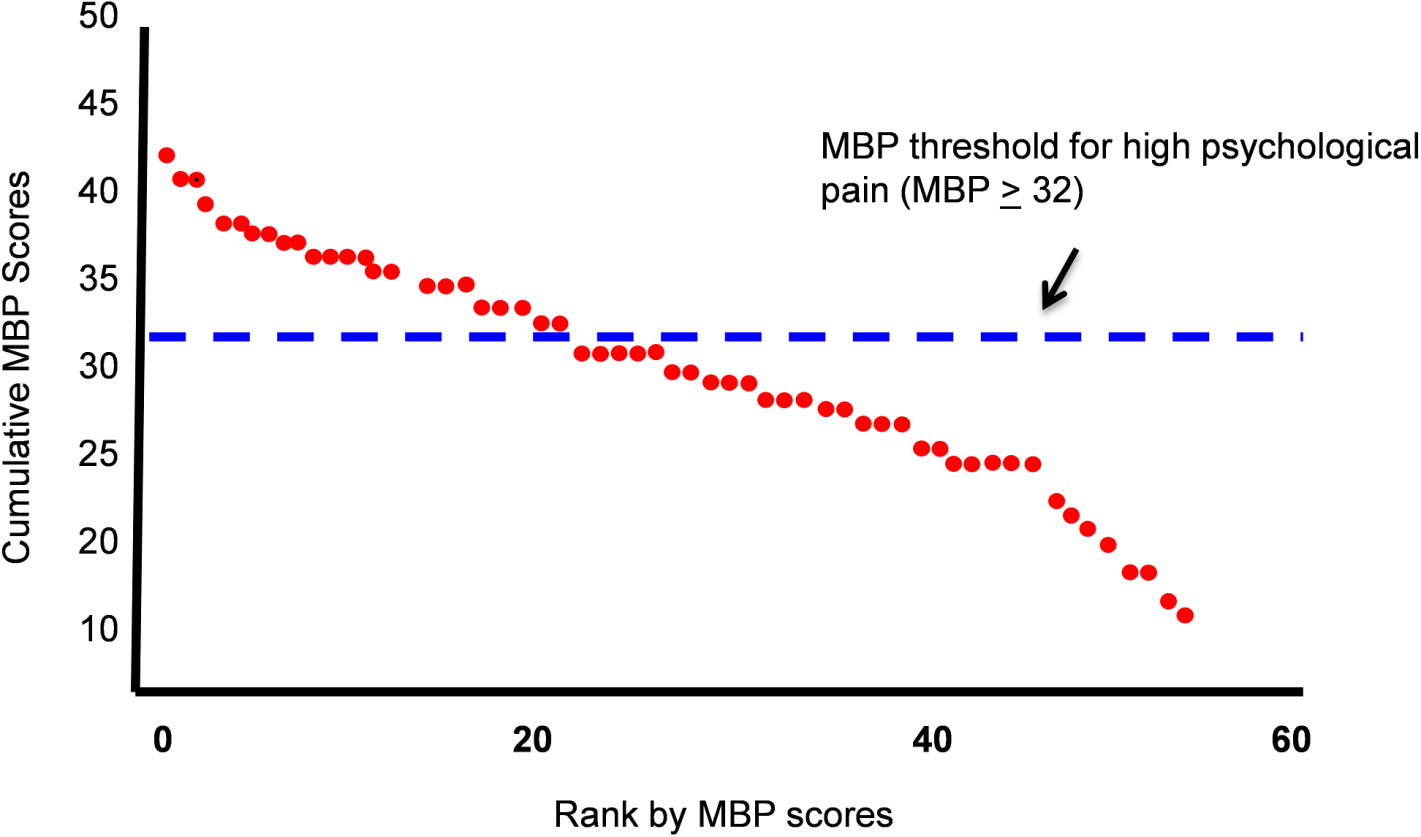
Cumulative distribution graph of MBP scores for suicidal patients (N = 57). Suicidal patients were ranked according to MBP scores from highest to lowest.

**Psychological pain (MBP):** 24/57 (42.1%) suicidal patients scored above a pre-determined threshold [5] for high psychological pain (MBP ≥ 32). (Mean_MBP_ suicidal _=_ 30.14 ± 7.43; Mean_MBP_ controls = 12.7 ±4.5, p>.001).(Fig 1).

**Beck Depression Inventory (BDI-II):** Overall depression scores were elevated (BDI mean = 13.2 ± 7.82. (BDI ranges: 0–9 minimal depression; 10–18 mild depression, 19–29 moderate depression, 30–63 severe depression).

**Beck Hopelessness Scale (BHS)**: Mean_BHS_ = 10.11 ± 6.90, considered to be moderate in intensity (Range: 0–3: none or minimal; 4–8: mild; 9–14: moderate may not be in immediate danger but needs monitoring; 15+: severe, definite suicide risk.

**Barratt Impulsiveness Scale (BIS):** Impulsiveness was elevated in suicidal patients (BIS mean = 74.12 ± 12.40). [Control scores are in the range of 50 to 60 [18]].

### Regression of C-SSRS on MBP, BIS, BDI and BHS

To determine the relationships between measured psychiatric constructs and suicidality, C-SSRS scores were independently regressed on each predictor in seven separate analyses. Based on the linear regression (Table 3), psychological pain (MBP) accounted for the largest proportion of variance in the C-SSRS, followed by overall impulsiveness level (BIS total score), depression (BDI), and hopelessness (BHS). Impulsiveness (BIS) and psychological pain (MBP) contributed more to suicidality than any of the other combined assessments. The additional variance in suicidality (C-SSRS) contributed by the MBP on top of what impulsiveness (BIS) explains alone is 10%. The additional variance contributed by depression (BDI) and hopelessness (BHS) did not reach statistical significance.

**Table 3:** Simple linear regression of psychological pain (MBP), impulsiveness (BIS), depression (BDI) and hopelessness (BHS) on suicidality (C-SSRS).

BLOCK 1					BLOCK 2^a^		
Rating Scale	Slope (s.e.)	r^2^	‘t’ (two-tailed)	p-value	Change in *r*^*2*^ *(Δr*^*2*^*)*	Total *r*^*2*^	p-value^b^ for *Δf*^*2*^
Psychological pain (MBP^c^)	0.328(0.79)	.24	4.14	< .001	n/a*	n/a	n/a*
Impulsiveness (BIS total^d^)	0.168 (0.047)	.19	3.60	< .001	.10	.29	.009
Depression (BDI^e^)	0.267 (0.078)	.17	3.40	.001	.08	.25	.012
Hopelessness (BHS^f^)	0.291 (0.90)	.16	3.24	.002	.09	.25	.021

^a^There are no values for MBP in Block 2 since these blocks represent additional contributions of MBP (i.e., change in *r*^*2*^***)*** to what the predictors in Block 1 contributed.

^b^Since multiple statistical significance tests were performed, the Type I error rate for all analyses was set at .01.

^d^Mee-Bunney Psychological Pain Scale

^e^Barratt Impulsiveness Scale

^f^Beck Depression Inventory

^g^Beck Hopelessness Scale

^c^Columbia Suicide Severity Rating Scale.

### Differentiation of patients scoring above and below the MBP threshold

In our previous study we used a predetermined threshold of MBP ≥ 32 representing 0.5 SD above the mean for depressed MDE patients as a cutoff for high psychological pain [5]. As illustrated in Table 4, patients ranking above threshold also scored significantly higher on the other assessments (C-SSRS, BDI, BHS, BIS) suggesting that these patients were more suicidal, depressed, hopeless and impulsive than patients with lower scores.

**Table 4:** Two-tailed ‘t’ tests showing significant differences in test scores between high and low scoring patients on the MBP^a^.

Rating Scale	Low psychological pain (MBP<31)	High psychological pain (MBP ≥ 32)	t-test	p-value
	N = 33	N = 24		
	Mean (SD)	Mean (SD)		
BIS^b^	69.83 (10.60)	80.95 (13.77)	-3.43	**< .001**
BHS^c^	7.71 (6.15)	13.91 (6.39)	-3.65	**< .001**
BDI^d^	10.20 (6.72)	18.05 (7.09)	-4.20	**< .001**
C-SSRS^e^	13.49 (4.82)	17.91 (4.06)	-3.58	**< .001**

^a^Mee-Bunney Psychological Pain Scale

^b^Barratt Impulsiveness Scale

^c^Beck Hopelessness Scale

^d^Beck Depression Inventory

^e^Columbia Suicide Severity Rating Scale

### Incidence of serious suicide events (SSEs) at a 15-month follow-up

At a 15-month follow-up we recorded 9 SSEs, including one completed suicide (S1 Table). Two additional patients made what were determined to be non-serious gestures. Seven of the nine patients rated above-threshold for high psychological pain while two patients making suicide gestures scored in mid-range. The SSE patient with the highest score (MBP = 42) overdosed on drugs within 36 days of assessment and had to be intubated, requiring hospitalization in the ICU. Another patient scoring above threshold (MBP = 39) had an interrupted suicide attempt throat slitting (within 2.5 months of assessment). The patient who completed suicide had dropped out of the VA program and had an above threshold score (MBP = 33). Two other SSE patients had high but slightly below-threshold scores of 30 and 31, respectively. There were no other suicide events.

Logistic regression did not identify any baseline variables that predicted future SSEs. This is likely a consequence of the small number of subjects having a serious suicide event combined with a relatively low variability in scale scores. Examination of MBP scores categorically (MBP <32; MBP ≥ 32) did however suggest a relationship with future SSEs (Chi-squared = 5.58, df = 1, p = .018) but did not reach significance since we are using a Type 1 error rate of 0.1.

### MBP scores in suicidal vs major depressive episode (MDE) patients

We compared MBP scores from suicidal patients and the cohort of MDE patients from our earlier study[5]. A post-hoc t-test showed no significant difference in means between the groups: (mean MBP_suicide_.30.14 ± 7.82,) and MDE (mean MBP_MDE_ 28.7 ± 6.4); t = -1.21, p = .23).

## Discussion

The primary focus of this study was to evaluate psychological pain in a US veteran population of suicidal patients with a recent (within 3 months) history of suicidality. Using the MBP scale we identified a subgroup (24/57) of patients meeting criteria for high psychological pain. These patients also scored significantly higher for depression (BDI), hopelessness (BHS), impulsiveness (BIS) and suicidality (C-SSRS) compared to patients with lower MBP scores suggesting that suicidal patients with high psychological pain may represent a more severely ill subgroup. Our findings expand on our original research in which we showed a significant linear correlation between psychological pain and suicidality in major depressive episode (MDE) patients [5].

The MBP had the highest predictive value for suicidality (as measured by the C-SSRS) than depression (BDI), hopelessness (BHS) and impulsiveness (BIS) in suicidal patients. These results are compatible with our findings in MDE subjects and are supported by a comprehensive review in which mental pain was found to be a stronger factor of vulnerability to suicidal ideation than depression [8].

Despite the growing number of investigations on psychological pain, the link between high levels of psychological pain and suicidality has not been firmly established. Within our suicidal cohort, less than half (42%) scored above threshold on the MBP. This may be due to the potentially transient nature of psychological pain where intense pain levels fluctuate [19] and as reported, may rapidly (within days) decrease [9].

Based in part on Shneidman’s hypothesis that unbearable psychological pain is a motivating factor for suicide [6], we expected that suicidal patients would have a significantly higher mean score on the MBP than MDE patients from our previous study [5]. However, a post-hoc analysis revealed no significant difference between the depressed and suicidal patients although we observed a trend for higher psychological pain in the suicidal group.

### Inpatients versus outpatients

No significant differences were found between in and outpatients on any of the evaluations although there was a non-significant trend for an increase in depression in the hospitalized patients.

Our data suggest that the weeks to months following high psychological pain could represent an increased period of vulnerability for a serious attempt. These results are consistent with previous studies showing a marked increase in suicides following discharge from psychiatric hospitals [20–23]. We identified 9/57 (15.8%) patients with serious suicide events including one completed suicide. All 9 patients had high (n = 7) or near high threshold (n = 2) psychological pain. The one completed suicide scored above threshold on the MBP. Two additional patients with low to mid-range scores on the MBP made non-serious suicide gestures. These data, although preliminary, suggest the potential importance of assessing psychological pain in high-risk populations to help target individuals for additional interventions.

Research is needed to identify the factors that contribute to the transition from suicidal ideation to an SSE. Our research suggests that high levels of pain may help facilitate the transition and perhaps, as suggested by Meerwijk et al., [19, 24] when tolerance to pain falls below a critical level, the risk for suicidality increases. When combined with depression, hopelessness and impulsiveness the risk may be further elevated [9–12, 25]. Our observation that lethality of the attempts were associated with high scores for psychological pain may be due in part to the MBP test items capturing additional aspects of suicidality including tolerance to psychological pain and reporting that dying was an option to make the mental pain stop. However, studies in larger populations are needed.

Comprehensive data collected on military personnel support recent social stressors (within 90 days of the event) as a contributing factor to suicidality. The Department of Defense Suicide Event Report (DoDSER)[26] identified major social stressors (e.g., failed intimate relationships, financial problems and administrative/legal issues) as probable contributors to completed suicides. Only one of the SSE patients in our study reported a social stressor (breakup with spouse) at the time of admission into the study. Possibly relevant is that this patient completed suicide by overdose and rated psychological pain on the MBP above threshold. Future studies could help define the interaction of psychosocial stressors and psychological pain as they relate to suicide risk.

There are several limitations to our study. Medication could not be controlled or ethically withheld and our patients represented a diagnostically heterogeneous suicidal population including MDD, BPD, and PTSD, some with co-morbidity for substance abuse. Finally, we included both in and outpatient subjects in analyses.

## Conclusions

A subset of patients with an increased vulnerability for experiencing high levels of psychological pain may represent a novel pathophysiological subtype of psychiatric disease with an increased risk for suicidality. Rather than solely focusing on the singular presence of suicidality, for example, proactively assessing underlying sub-constructs of suicide such as psychological pain, impulsiveness and hopelessness carries the potential for improving the identification of high-risk individuals.

Although many of the tests for suicidality including the Beck Hopelessness Scale (BHS), Beck Depression Scale (BDI) and Suicide Intent Scale (SIS)] have increased sensitivity for suicidality, they may not be as accurate for suicide prediction [27]. The addition of assessment tools such as the MBP to measure psychological pain may help further delineate a high-risk subpopulation for increased clinical interventions. Since a high proportion of suicides occur within close temporal proximity to clinician visits [28] and discharge from psychiatric hospitalization [20, 29], assessment with a combination of measures may improve risk evaluation during this important period.

## Supporting information

S1 Table Clinical descriptors of patients with serious suicidal events (15 month follow-up). (DOCX)
