# Supplementary material for: Assessment of psychological pain in suicidal veterans
Source: PLoS One. 2017 May 30;12(5):e0177974. doi: 10.1371/journal.pone.0177974 (PMC5448740; doi:10.1371/journal.pone.0177974)
Supplement: S1 Table — (DOCX) [file pone.0177974.s001.docx]

**SUPPORTING INFORMATION**

**TABLE 1: Clinical descriptors of patients with serious suicide events (15-month follow-up)**

| **Pt** | **Reason for referral** | **Sex** | **Age (yrs)** | **Ethnicity** | **Status at admission** | **Diagnosis** | **MBP score at admission into study** | **Time from evaluation to event** | **Serious suicidal behavior** |
| --- | --- | --- | --- | --- | --- | --- | --- | --- | --- |
| 1 | 3 past suicide attempts | M | 56 | Caucasian | Outpatient | BPD, polysubstance abuse | 35* | 7 days | Cutting throat with scissors |
| 2 | Suicide attempt with Vicodin overdose after break-up with spouse | M | 51 | Caucasian | Inpatient | MDD, alcohol dependence | 33* | 210 days | Completed suicide by overdose |
| 3 | Suicidal ideation plus plan  Fixed delusions of brain monitoring | M | 50 | Caucasian | Inpatient | Depressive disorder NOS, stimulant-induced psychosis, PTSD | 40* | 57 days | Hearing voices telling patient to “die by breathing in car exhaust (“I am just tired of life and want to end it all.”) |
| 4 | Attempted hanging  (“I would be better off dead”) | M | 22 | Hispanic | Inpatient | PTSD, alcohol dependence, depressive disorder NOS, borderline PD | 42* | 36 days | Drug overdose-required intubation and ICU stay |
| 5 | Suicidal ideation with plan. Trying to get hit by trucks walking on side of road; past history of suicide attempts | M | 51 | Caucasian | Outpatient | BPD, PTSD | 35* | 180 days | Found walking on railroad tracks, stating “nothing to live for” and “it would soon be over” |
| 6 | Plan to hang self in 2 days | F | 48 | Black | Outpatient | BPD, PTSD, borderline personality disorder | 33* | 420 days | Acute suicidal ideation, feels like “can’t keep from suicide” |
| 7 | Past history of suicide attempts. Suicidal ideation with plan to overdose | M | 61 | Hispanic | Inpatient | BPD, PTSD | 39* | 75 days | Interrupted suicide attempt to cut throat |
| 8 | Threatened to jump in front of car, past suicide attempts | M | 44 | Caucasian | Inpatient | BPD, PTSD | 31 | 16 days | Attempted suicide with 45 Tylenol, 35 sleeping pills plus alcohol |
| 9 | Suicidal ideation with plan | M | 25 | Asian | Inpatient | MDD, Alcohol abuse | 30 | 57 days | Tried to jump off bridge |

PTSD=post-traumatic stress disorder; MDD=Major depressive disorder; BPD=bipolar disorder; NOS=not otherwise specified

*MBP scores above threshold for high psychological pain
